# The Effects of Feeding Unpredictability and Classical Conditioning on Pre-Release Training of White-Lipped Peccary (Mammalia, Tayassuidae)

**DOI:** 10.1371/journal.pone.0086080

**Published:** 2014-01-27

**Authors:** Selene S. C. Nogueira, Shauana A. Abreu, Helderes Peregrino, Sérgio L. G. Nogueira-Filho

**Affiliations:** 1 Laboratório de Etologia Aplicada, Universidade Estadual de Santa Cruz, Ilhéus, Bahia, Brazil; 2 Laboratório de Fisiologia do Comportamento, Universidade Federal do Rio Grande do Norte, Natal, Rio Grande do Norte, Brazil; Institut Pluridisciplinaire Hubert Curien, France

## Abstract

Some authors have suggested that environmental unpredictability, accompanied by some sort of signal for behavioral conditioning, can boost activity or foster exploratory behavior, which may increase post-release success in re-introduction programs. Thus, using white-lipped peccary (*Tayassu pecari*), a vulnerable Neotropical species, as a model, we evaluated an unpredictable feeding schedule. Associating this with the effect of classical conditioning on behavioral activities, we assessed the inclusion of this approach in pre-release training protocols. The experimental design comprised predictable feeding phases (control phases: C_1_, C_2_ and C_3_) and unpredictable feeding phases (U_1_- signaled and U_2_- non-signaled). The animals explored more during the signaled and non-signaled unpredictable phases and during the second control phase (C_2_) than during the other two predictable phases (C_1_ and C_3_). The peccaries also spent less time feeding during the signaled unpredictable phase (U_1_) and the following control phase (C_2_) than during the other phases. Moreover, they spent more time in aggressive encounters during U_1_ than the other experimental phases. However, the animals did not show differences in the time they spent on affiliative interactions or in the body weight change during the different phases. The signaled unpredictability, besides improving foraging behavior, showing a prolonged effect on the next control phase (C_2_), also increased the competition for food. The signaled feeding unpredictability schedule, mimicking wild conditions by eliciting the expression of naturalistic behaviors in pre-release training, may be essential to fully prepare them for survival in the wild.

## Introduction

The white-lipped peccary (*Tayassu pecari*) is a Neotropical ungulate that is categorized as a vulnerable species due to overhunting and deforestation [1]. Because of that, some herds of white-lipped peccary have been kept in captive breeding centers to replace stocks lost due to hunting or deforestation impacts [1], [2], [3]. To date, however, no pre-release training program has been carried out with this species. The inclusion of environmental enrichment schedules in pre-release programs can develop specific skills in captive animals that may improve their survival in the wild [4]. An enrichment program may improve the captive animals' welfare or their allostasis - stability through change [5] -, which is related to their biological functioning; thus, an animal in good shape in terms of welfare is also in good shape to face environmental challenges. In addition, the behavioral conditioning technique has also been shown to be an important tool for reinforcing desirable behaviors to prepare animals for release into the wild [6], because animals can learn how to perform behaviors that increase their fitness and are important for reproductive success [7].

Few studies, however, provide a better understanding of the benefits of an unpredictable schedule for captive peccaries, with a view to preparing them to be released in the wild. These approaches are important because in natural conditions peccaries have a vast home range area, up to 100 km^2^, where they are almost constantly involved in foraging and roaming up to 13 km per day [8]. Otherwise, in zoos and other conservation centers they are kept in paddocks ranging from 400 m^2^ to 8,000 m^2^ and receive food once or twice a day in a predictable routine schedule [9], decreasing their exploratory skills. The maintenance of these wild animals in such restrictive environments can change their behavior [10], due to reduced availability of space and food variability, leaving them ill-equipped to respond appropriately to natural stimuli, as observed in other species, such as African wild dogs (*Lycaon pictus*) and black rhinoceros (*Diceros bicornis*) [11], [12], [13]. Thus, the predictable captive environment may compromise *ex situ* and *in situ* conservation efforts, because of deleterious effects on individual survival and reproduction success after the animals are released in the native habitat [14], [15].

Despite some predictable characteristics found in the wild, such as seasons and time of day, it is also important to consider unpredictable aspects in this environment, including finding food and refuge or facing a predator attack. In this context, it is vital in pre-release programs to take unpredictability into account to prepare peccaries for the unexpected, thereby mimicking wild conditions. Thus, animals' cognitive skills are developed [16], [17], [18], and they are consequently better able to survive in the wild environment, because their exploring and foraging capabilities will have been honed [11].

Suggesting a theory on the effects of environmental enrichment, Watters [19] highlights the potential benefits of environmental unpredictability for captive animals, which could improve their wellbeing and consequently favor the animals' survival after release into the wild. A brief literature survey on the benefits for captive animals maintained in an unpredictable environment makes it clear that this issue is controversial. For some species, environmental unpredictability provides positive effects [20], [21], such as boosting foraging and exploratory behaviors in coyotes (*Cannis latrans*) and collared peccaries (*Pecari tajacu*) [22], [23]; while for others it results in negative effects, because certainty may cause more confidence, for example in brown capuchins (*Cebus paella*) avoiding anticipation behaviors [24]. This difference may be explained by natural environmental characteristics *per se*, because in the wild, the animals experience both predictability and unpredictability. Under predictable signals (like day and night), animals may experience more confidence by predicting some events, while unexpected events may make the environment more stimulating, promoting more motivation and preventing boredom and depressive behaviors, which can compromise animals' welfare and consequently jeopardize conservation programs. So, in captivity, adding a reliable signal (predictable), using classical or operant conditioning [7], [24], [25] during an unpredictable program, may improve animals' confidence while triggering positive behaviors [13], [22], [26].

Thus, the aims of this study were to evaluate the effects of non-signaled and signaled food (classical conditioning) during an unpredictable feeding schedule, so as to develop a pre-release training protocol for white-lipped peccaries. This in turn aimed to elicit the expression of naturalistic behaviors and maintain fitness skills, which can be essential for survival outcomes [27]. We predicted that by adding a signal during feeding unpredictability, using classical conditioning, the peccaries would become more active by increasing exploratory behaviors.

## Materials and Methods

### Ethics Statement

This work followed the “Principles of laboratory animal care” (NIH publication No. 86-23, revised 1985) and was approved by the Committee of Ethics for Animal Use (CEUA) at the Universidade Estadual de Santa Cruz (protocol #003/07).

### Animals and Housing

**Figure 1:**
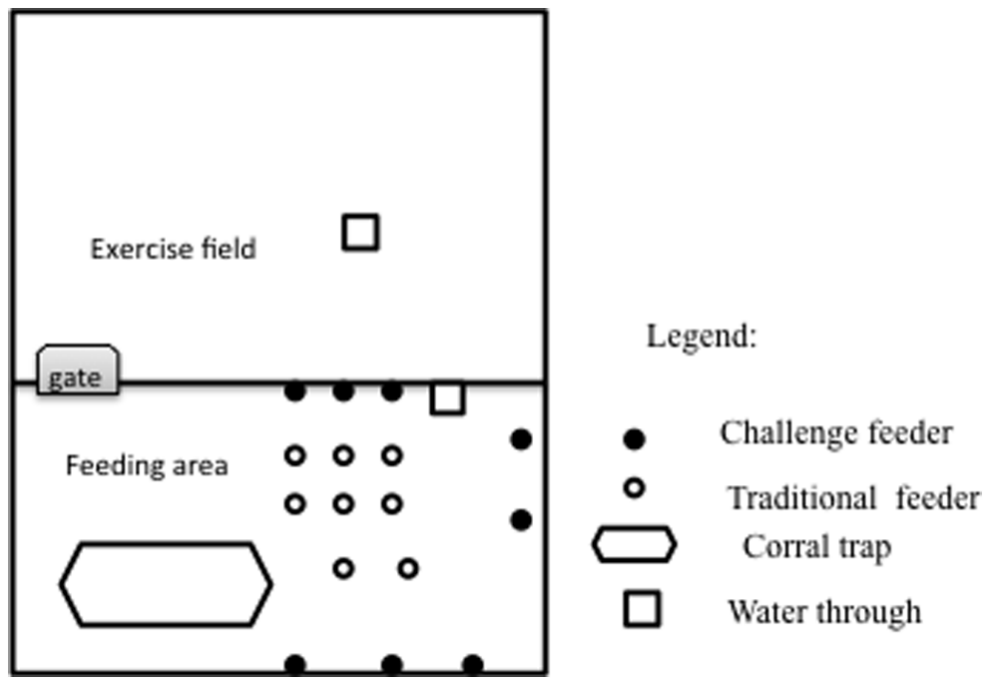
The paddock design, comprising exercise field and feeding areas.

Twelve white-lipped peccaries, six males and six females, all adults aged between four and six years old and weighing in average (± SD) 34.2 (±4.0) kg, were observed at the Laboratório de Etologia Aplicada (LABET), Universidade Estadual de Santa Cruz-UESC, Ilhéus, Bahia, Brazil (14°47′39.8″S, 39°10′27.7″W). All animals were born and raised in captivity. The animals were individually identified with plastic ear tags of different shapes and colors. They were housed in an experimental paddock (940 m^2^), which represented a space allowance of *c.a.* 80 m^2^ per animal. This area was divided in two by a wire fence with a gate: exercise field (564 m^2^) and feeding area (376 m^2^) (Fig. 1). The feeding area contained a corral trap (10.0 m long×9 3.0 m wide) used for animal management when necessary (Fig. 1). Both spaces contained dirt floors; the feeding area had much less vegetation, while the exercise field contained high and medium-sized trees, their branches providing spots with natural shade and hiding areas. A 1.5 m-high wire netting fence surrounded the paddock, and both areas (exercise field and feeding area) contained one water trough (0.6 m length×0.3 m breadth).

We adopted the same unpredictable feeding method used for collared peccary [23], applying traditional feeders (TF) and challenge feeders (CF). The TFs were the feeders regularly used in LABET, while the CFs are new feeders designed for collared peccaries [23] (details below), which encourage animals to put some work into opening them. The TF were made from truck tires cut in half and laid horizontally (1.0 m length×1.0 m breadth×0.3 m height). These feeders are usually chosen by farmers because they are cheaper than building concrete ones. Eight traditional feeders (TFs) were available in the feeding area; however, we fed the animals in just four feeders, which were always the same and were also located in the same spot during the control phases (C). The CFs, used during the unpredictable feeding phases (U: described below), followed the same design and dimensions employed for collared peccaries [23]. They were made with 1.0 m-long PVC tubes (diameter 150 mm) and fitted with a sprung door (0.30 m height×0.15 m breadth). The CF design mimics the peccaries' natural foraging, using their snouts to catch worms or eating roots [28]. To reach their feed, the peccaries needed to use their snouts to open the feeder doors, which then closed immediately when the animals withdrew their heads.

At all times we provided one feeder for every three animals, since this was the same throughout the experimental phase, placing them 5.0 m apart, based on the results obtained for collared peccary [29]. Prior to starting the study, the animals were fed twice a day, at 10:00 h and 16:00 h. The diet comprised a mixture of maize grain, soybean meal, and mineral salts, providing 12% crude protein and 3,300 Kcal/kg of gross energy [30]. The feed was always pre-weighed, corresponding to 3.5% of live weight per animal on a dry matter basis, and then poured into the feeders. The diet remained the same before and during the experiment, and water was available *ad libitum*.

### Experimental design and Procedures

The experimental design followed the ABABA model [31]. The A phases represented the control phases (predictable: C_1_, C_2_, and C_3_), and the B phases represented the feeding unpredictable phases (U_1_-unpredictable signaled and U_2_- unpredictable non-signaled). During U phases (U_1_ and U_2_), the unpredictability was applied by using eight CFs plus spatial and temporal unpredictability. On the other hand, during control (C_1_, C_2_, and C_3_) phases, we used the TF plus spatial and temporal predictability, e.g. same feeding time and location.

Each phase (C_1_, U_1_, C_2_, U_2_, and C_3_) lasted 10 days. During control or predictable phases, feed was offered in the usual TF. During unpredictable phases feed was offered in the CF. The CFs received a number for later identification during the random choice unpredictable phases. Prior to feeding times and data collection, all eight CFs were removed from the feeding area and checked for any feed remains, and were then put back in the feeding area in the same positions. Of these eight CFs, only four of them contained enough feed for three animals, a quantity of 1.2 kg, resulting in the same protocol as that employed for the TF. This was to provide spatial and temporal unpredictability for the animals during the phases (U_1_ and U_2_). Additionally, before each session started, we randomly chose which CFs were filled, by drawing lots (Fig. 1). Therefore, the animals had to search for food among the eight feeders. During the first unpredictable phase (U_1_), we added a whistle signal by using a plastic whistle to condition animals to come over to the feeding area, exactly when feed was provided. Throughout U_1_ and U_2_ phases the feed was offered twice daily, following the same protocol adopted in the control phases. However, the two feeding times were randomly chosen by a draw of times between 8:00 h and 17:00 h, following the normal routine of peccaries in captivity and not at fixed times as in the controls (C), presenting the animals with a level of uncertainty.

### Training for Whistle Signal and Challenge Feeder

The animals were habituated to remaining in the exercise field area, where they stayed most of the time except during feeding. Ten days before phase C_1_ started, the animals were conditioned to get food after hearing a whistle signal and then entering the feeding area. Seven days after training started the animals were completely trained to come and get food when the keeper signaled with the whistle. This classical conditioning training was done while animals were using the traditional feeders (TF-described above), before application of the unpredictable feeding schedule. In addition, the caretaker was instructed to go to the feeding area at unpredictable times for inspections, without any signal. This procedure was carried out for a month before the study started, so that the animals would not associate caretaker movement with food delivery. We always proceeded this way during the entire experiment, before feeding time and twice more during the day, trying to mask feed delivery cues and not raising animals' expectations of feed.

To train animals to answer the whistle command, first the caretaker filled the traditional feeders (TF) outside the feeding area, out of the animals' sight. Afterward, the caretaker allocated the feeders inside the feeding area and immediately whistled three times, opening the gate by using a rope for animals to enter the feeding area.

Following the whistle conditioning, the peccaries were trained to open the CF doors for three days. On the first day, the feeders were filled and the doors remained completely open for two hours in the morning and in the afternoon. On the second day the challenge feeders were filled and the doors left halfway open, and approximately three hours later all animals had successfully accessed feed. On the third day the feeder doors were completely closed. One hour later, the peccaries had worked out how to open the doors. By the end of the 10 days, all individuals not only came when they heard the whistle command but were also able to open the CF doors.

### Behavioral Data Collection

We habituated the peccaries to the presence of an observer 10 days before the data collection. The observer recorded the data from an observation spot located outside the paddock fences. We randomly chose the order in which animals were observed, before the observations began. Each focal animal's activities [32] were recorded for five continuous minutes with digital camcorders (DCR-SR45 Sony, Tokyo, Japan). The peccaries' behavioral patterns for feeding, exploratory, inactive (resting), aggressive, and affiliative moments were categorized. The aggressive patterns included chase, attempt to bite and threat, while the affiliative ones included mutual rubbing and grooming [9]. All individuals were continuously visible during the behavioral data collection. We observed all the peccaries for one hour on the same days in at least one of the two feeding periods. Therefore, each animal was recorded for 5 minutes/observational session, totaling 60 minutes per individual in each experimental phase.

### Animals' Weight Change

Five days before each experimental phase began (C_1_; U_1_; C_2_; U_2_ and C_3_) we individually restrained the peccaries with a net to weigh them and thus evaluate the animals' weight change. Such procedures normally cause animals distress during the following four days [33], but from five days onwards the peccaries show normal behaviors. Thus, for the effects of distress caused by restraining not to impinge on the following experimental phase, observations started just five days after the animals had been weighed before each experimental phase. Despite this gap in observations all phases ran consecutively. We also weighed all individuals at the end of each phase, so the weight change was calculated by difference.

### Data Analyses and Statistical Methods

The digitized behavioral records were analyzed by using Ethoplayer 1.3 software (Leo Software Inc., Toulouse, France), which provides an electronic stopwatch that allowed the observer to record the time spent on each behavioral pattern by each focal individual. The time spent on each selected behavioral pattern from multiple days was totaled to create one record per individual in each control and experimental phase during feeding periods. Thus, the time spent on the selected behavioral pattern was compared among the different experimental phases.

We chose the ABABA experimental design [31], [34] and the following statistical analysis procedures [35] to avoid poor interpretation of data due to our limitation of studying only one white-lipped peccary group. Thus, we compared the time spent on each selected behavioral pattern by separate analysis of variance (ANOVAs) with repeated measures followed by *post hoc* Duncan test (Statistica version 7.0 - StatSoft, Tulsa, OK, USA), when appropriate. We included in the model the effects of the experimental phases (C_1_, U_1_, C_2_, U_2_, and C_3_) and the sex (male vs. female) as independent factors. Using this same model, we compared the weight change.

## Results

During the observation sessions the peccaries showed differences in the time spent on exploratory behaviors according to the experimental phases (F_4, 40_ = 10.03, *P* = 0.00001). The *post hoc* test showed that the animals equally explore more (*P*>0.16) during the unpredictable signaled (U_1_) and non-signaled (U_2_) spatial and temporal unpredictability phases and during the second control phase (C_2_) than during the other two control (C_1_ and C_3_) phases (Table 1), when feed was offered at the usual feeding time and location in traditional feeders (TF). Independently of the phases, males spent more time exploring than females (F_1, 10_ = 11.49, *P* = 0.006).

**Table 1:** Mean ± SD of the time (s) the white-lipped peccaries spent on exploratory, inactive, feeding, aggressive, and affiliative behavioral patterns plus the body weight change (g/day) during the experimental phases (C_1_U_1_C_2_U_2_C_3_, C: control and U: unpredictable).

Variables	C_1_	U_1_	C_2_	U_2_	C_3_
Exploratory	249.7±187.5^a^	571.1±242.9^b^	520.5±215.1^b^	550.9±195.8^b^	359.9±188.0^a^
Inactive	1370.0±586.7^a^	685.0±480.3^b^	730.0±225.7^b^	1080.0±191.5^ab^	1200.0±144^a^
Feeding	1086.0±493.4^a^	696.5±219.0^b^	693.9±284.6^b^	971.4±384.3^a^	1017.2±457.0^a^
Aggressive	1.6±1.9^a^	6.6±6.3^b^	2.1±3.8^a^	3.1±3.8^a^	1.5±4.6^a^
Affiliative	43.9±25.9^a^	70.6±45.8^a^	83.3±93.4^a^	70.1±93.6^a^	133.1±168.8^a^
Body weight change	8.0±19.5^a^	15.5±18.7^a^	18.0±16.9^a^	17.4±23.1^a^	24.5±23.1^a^

Different superscript letters in the same line correspond to significant differences (*P*s<0.05).

The peccaries also showed differences in the time they showed inactivity during the observation sessions among experimental phases (F_4, 40_ = 8.29, *P* = 0.00006). The *post hoc* test showed that the peccaries were equally (*P*>0.13) inactive for smaller amounts of time during the signaled unpredictable phase (U_1_) and the second control phase (C_2_) than during the other two control phases (Table 1). There was a tendency (*P* = 0.067) for the peccaries to be more inactive during the non-signaled unpredictable phase (U_2_) than during the signaled unpredictable phase (U_1_, Table 1). Males and females did not differ in the amount of time they were inactive (F_1, 10_ = 0.32; *P* = 0.58).

Moreover, there were also effects on the time the peccaries spent feeding, showing differences among the experimental phases (F_4, 40_ = 2.96, *P* = 0.03). The peccaries spent equally less time feeding (*P*>0.98) during the signaled unpredictable phase (U_1_) and also the following control phase (C_2_) than during the non-signaled unpredictable phase (U_2_) and the other two control phases (C_1_ and C_3_, Table 1). Males and females did not differ in the time they spent feeding (F_1, 10_ = 3.25, *P* = 0.10). There were also effects on the time the peccaries spent in aggressive interactions, differing among the experimental phases (F_4, 40_ = 3.26, *P* = 0.02). The *post hoc* test showed that more competition (*P*s<0.04) occurred during the unpredictable signaled (U_1_) than during the other phases (Table 1), without differences between males and females (F_1, 10_ = 0.05; *P* = 0.83). Nevertheless, during the different phases, the animals did not show differences in the time they spent on affiliative interactions (F_4, 40_ = 1.60, *P* = 0.19, Table 1). Furthermore, there were no differences in the body weight change among the phases (F_4, 40_ = 1.17, *P* = 0.34, Table 1). Males and females also did not differ in these variables (F_1, 10_ = 0.08, *P* = 0.79, F_1, 10_ = 3.7, *P* = 0.09, respectively).

### Discussion

Unpredictability and the use of a signal increase peccaries' activity, while keeping animals at higher levels of exploratory behaviors. Such positive effects, however, were also observed during the non-signaled feeding unpredictability phase. Although no differences were noted between signaled and non-signaled phases, we observed a carryover effect of an increase in exploratory and feeding behaviors and a consequent decrease in inactivity during the control phase that followed the signaled phase. Such effects were not seen during the control phase that followed the non-signaled phase, suggesting that operant conditioning by whistle signaling was generalized [7] to the following control phase (C_2_).

Classical conditioning theory can explain these carryover effects on positive behaviors. This theory predicts that accompanied by conditioned stimulus (whistle) other environmental context cues can influence individual association [7]. As much as we tried to avoid external interference during conditioning training, some environmental cues were always present during the signaled phase. These cues included food odors, sounds, and caretaker movements that could represent *contextual stimuli* [7], which animals could learn, associate and use in the second control phase (C_2_), spreading positive effects even without whistle signal presence.

The carryover effects may also be related to anticipation of reward involving dopaminergic effects, since animals may predict feed delivery, experiencing pleasure [35]. Dopamine or endorphin properties have been associated with appetitive behaviors [36], motivating animals to continue exploring and consequently be more active, as observed in the present study. Authors [37] reported that rooting (exploratory behavior) in domestic pigs has the same rewarding positive characteristics as available food *per se*. In our study feeding behavior also showed a carryover effect on the time the animal spent on exploration, feeding and activity during the control phase after the signaled unpredictable phase. The obtained results, therefore, show that the adoption of feeding unpredictability associated with classical conditioning in pre-release training of white-lipped peccary could be very advantageous for the animals' survival after release into the wild. Because this method expands the positive effects of exploring the environment, it could equip animals to look for food in the wild. In a previous study, researchers [38] showed that non-trained peccaries re-introduced in a Savanna area located in Mato Grosso do Sul State, Brazil, remained dependent on the artificial source of energy – grain corn furnished in automatic feeders – instead of searching for natural food. The non-occurrence of carryover effect during the third control phase (C_3_), after the non-signaled feeding unpredictability one, could be explained by the extinction of animals' conditioned response. Here, extinction refers to the reduction in a conditioned response due to the lack of reinforcing consequences [7]. In our experimental design, however, the reinforcing consequences -to find and eat food - were present during all experimental phases, invalidating the extinction hypothesis. In contrast, the conditioned signal (whistle) was the only difference between both unpredictable schedules (U_1_ and U_2_). Thus, the non-occurrence of carryover effect during C_3_ reinforces the importance of using a conditioned signal for training peccaries to be released. This non-carryover response highlights the fact that animals can lose their exploratory motivation faster due to the non-presence of the associated signal in the unpredictable schedule, which can further prolong its effects. In the present study, besides exploratory behavior and activity effects, we observed that the peccaries spent less time feeding during the signaled unpredictable phase (U_1_) and the following control phase (C_2_) than during the other phases, suggesting that the conditioned signal reflected a safe signal for food, directing animals positively to consummatory behavior (eating food), resulting in their eating the same amount of food in less time. This will probably favor the animals' survival in the wild. Unfortunately, no comparable data are available for wild peccaries, but the decrease in the time spent feeding may be associated with an increase in awareness or caution. If it is true, this result is desirable in a pre-release program, because under natural conditions animals face environmental adversities, such as potential predators around them, which will require more awareness skills and consequently less time feeding in the same spot.

The increase in the time the peccaries spent in aggressive interactions can also explain the shorter time they spent on feeding during the signaled unpredictable phase (U_1_). The increased competition for food resources may also be related to anticipation of reward involving the dopaminergic effects mentioned above. Rather than being negative, the increase in competition leads to the development of varied skills and behaviors, which are necessary for survival outcomes [13]. Moreover, the proportion of time spent on competitive encounters was relatively low and we saw no aggressive interaction that have led to bleeding or wounds, leading us to consider this level of competition acceptable. Furthermore, there was no change in the occurrence of affiliative interactions among the experimental phases and it is also important to highlight that although the animals spent less time feeding during the phases U_1_ and C_2_, we did not find differences in the body weight changes when all phases were compared, which is also positive.

In addition, the conditioned signal could improve re-capture success during post-release monitoring. Previously, Figueira (personal communication) obtained low re-capture rates by using only automatic feeders to attract animals placed inside corral-traps used for monitoring the released population of peccaries. Therefore, in areas without poaching, the use of a signal may improve animal recapture, due to the animals' fast response in the direction of food.

White-lipped peccaries usually forage across huge areas, so in captivity we expected that most of the animals' natural activities, including exploratory behaviors, would decrease, contributing negatively to their welfare [39] and compromising both *in situ* and *ex situ* conservation efforts. The use of both signaled and non-signaled feeding unpredictability schedules elicits the expression of these naturalistic behaviors in pre-release training, which can be essential to post-release success by avoiding dependence on the artificial source of energy, as observed before among untrained peccaries [38]. The use of feeding unpredictability plus signal, however, resulted in a more prolonged effect and may improve re-capture success during post-release monitoring, in protected areas, due to the animals' fast response in the direction of food. Furthermore, the signal enhanced competition in the pre-release training, which could be essential to fully preparing them for life in the wild [39]. Therefore, the signaled feeding unpredictability schedule may be adopted in the pre-release training of this and other endangered species, such as the Chacoan peccary, *Catagonus wagneri*.
